# Use of Open Claims vs Closed Claims in Health Outcomes Research

**DOI:** 10.36469/001c.87538

**Published:** 2023-09-05

**Authors:** Onur Baser, Gabriela Samayoa, Nehir Yapar, Erdem Baser, Fatih Mete

**Affiliations:** 1 City University of New York, New York, NY, USA; 2 University of Michigan, Ann Arbor, Michigan, USA; 3 John D. Dingell VA Medical Center, Detroit, Michigan, USA; 4 Columbia Data Analytics, New York, New York, USA; 5 Mergen Analytics, Bilkent Cyberpark, Ankara, Turkey

**Keywords:** open claims, closed claims, MarketScan®, PharMetrics® Plus, Kythera, outcomes research

## Abstract

**Background:** Closed claims are frequently used in outcomes research studies. Lately, the availability of open claims has increased the possibility of obtaining information faster and on a larger scale. However, because of the possibility of missing claims and duplications, these data sets have not been highly utilized in medical research. **Objective:** To compare frequently used healthcare utilization measures between closed claims and open claims to analyze if the possibility of missing claims in open claims data creates a downward bias in the estimates. **Methods:** We identified 18 different diseases using 2022 data from 2 closed claims data sets (MarketScan® and PharMetrics® Plus) and 1 open claims database (Kythera). After applying an algorithm that removes possible duplications from open claims data, we compared healthcare utilizations such as inpatient, emergency department, and outpatient use and length of stay among these 3 data sets. We applied standardized differences to compare the medians for each outcome. **Results:** The sample size of the open claims data sets was 10 to 65 times larger than closed claims data sets depending on disease type. For each disease, the estimates of healthcare utilization were similar between the open claims and closed claims data. The difference was statistically insignificant. **Conclusions:** Open claims data with a bigger sample size and more current available information provide essential advantages for healthcare outcomes research studies. Therefore, especially for new medications and rare diseases, open claims data can provide information much earlier than closed claims, which usually have a time lag of 6 to 8 months.

## INTRODUCTION

An individual’s specific needs and desired health outcomes are the driving forces behind all healthcare decisions and quality measurements. Collecting data that clearly guide patients’ actions along their healthcare journey is crucial for health economics and outcomes research (HEOR) companies to provide patient-centered care.

Randomized clinical trials (RCTs) can answer many questions concerning treatments and health care. However, the volume of clinical questions greatly outweighs the available resources to conduct RCTs. A number of other limitations are also associated with RCTs.^1^ The results of an RCT might not reflect the effects of the treatment in real-world settings because they often assess efficacy in controlled, standardized, and highly monitored settings and usually among a highly selective sample of patients.^2^ Adherence or overdose are not at issue in patients in RCTs. In contrast, real-world evidence (RWE) does not have these limitations; therefore, there is a growing interest among regulatory and payor organizations to use nonrandomized RWE to supplement RCT evidence and aid in clinical and economic decision-making.^3^

RWE studies focus on the benefits and harms of treatments using real-world data; that is, routinely collected data relating to patients health status and/or the delivery of health care.^3^ Sources of real-world data, such as health insurance claims, electronic health records (EHR), and patient registries, are becoming increasingly consolidated, standardized, and accessible for research on therapeutics. RWE studies relying on existing data can often be implemented more rapidly than RCTs, providing an important advantage in the context of a new, rapidly spreading disease with high morbidity and mortality.

Real-world data can capture hospitalizations, causes of death, medication dispensing, and tests performed. Measurement of dispensed outpatient medication is typically well-captured through pharmacy claims.^4^ An exception is out-of-hospital deaths, which are captured poorly in data sources not linked to administrative or other death records. Also, while test results are also not typically recorded in claims, a positive result may be inferred from a test followed by an *International Classification of Disease, Tenth Revision* (ICD-10) diagnosis code.

Two kinds of data collection are available when providing RWE of a patient’s care journey: closed-payer claims and open claims. Outcomes research studies have typically used closed-payer claims for RWE. Open claims, on the other hand, are currently not commonly used in outcomes research but have lately risen in prominence. The utilization of open- and closed-payer claims is crucial for comprehending the current condition and undertakings of patients throughout their healthcare journey. Both data types offer distinct advantages for outcomes research companies to assess the economic effectiveness of recently introduced medication, study rare diseases, or enhance the recruitment process for clinical trials. This article will describe open and closed claims and compare some of the healthcare utilization values derived from both types.

### Closed Claims vs Open Claims

Claims data refer to the information derived from the processing of a healthcare claim. While claims are handled primarily for the purpose of payment, the data obtained from these claims are also utilized for secondary healthcare research.

Closed-payer claims data refers to information from payers that can be provided directly by health insurance companies or a collection of employers sharing their employees’ health claims with consulting services, revealing nearly all of a patient’s healthcare activities within a fixed period of enrollment. PharMetrics® Plus, MarketScan®, Optum, and Premier are some of the examples of commercial closed claims databases. Medicare, Medicaid, Veterans Affairs, and Department of Defense data sets are examples of government closed claims databases.

The data are generated directly from the insurer. Therefore, when patients visit different doctors, hospitals, and testing centers, all these actions can be captured if patient is enrolled with a healthcare insurer. Over-the-counter medications and medications paid for in cash may not be captured in the closed claims. With the enrollment file and eligibility information, the data also reveals when a patient does not visit the doctor or fill a prescription; therefore, adherence to treatment can be estimated.

**Figure 1.**
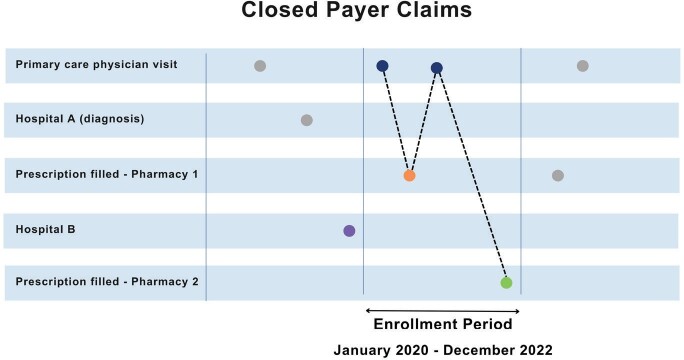
Patient’s Journey in Closed-Payer Claims

Suppose Patient A is enrolled for health coverage between January 2020 and December 2022 and sees a primary care physician, visits two different hospitals, and has a prescription filled from two different pharmacists. However, the diagnosis date was prior to enrollment in that insurance plan. We can see a comprehensive view of patient’s actions through closed claims (**Figure 1**).

In all settings of care, closed payers’ claims can provide a robust timeline of all events, which is critical for many types of outcomes research studies. Therefore, these types of claims have been used as a standard for RWE.

Despite their advantages, closed claims data have some drawbacks. First, most outcomes research looks for continuous care, which requires continuous enrollment of patients for the analysis. Although continuous enrollment is not typically a problem with government claims such as Medicare, it does decrease the sample sizes significantly for commercial claims. The average person in the US changes their insurance provider regularly. Approximately 1 in 5 members is disenrolled from a commercial insurer each year, and, on average, patients change insurers every 18 months.^5^ Sixty-five percent of Americans change jobs within 3 years.^6^ These changes directly affect sample size. The total sample size for MarketScan® closed claims, which receives its information from health claims shared by employers, decreased from 39 million to 17 million with a 3-year continuous enrollment requirement; PharMetrics® closed claims, which receives its information mainly from one of the biggest insurance companies in the US, decreased from 45 million to 14 million with a 3-year continuous enrollment requirement. Since most RWE studies require 1-year identification periods, a 1-year pre-index period, and a 1-year post-index period, this could result in a significant decrease in diseases diagnosed or medications dispensed where there is a small number of patients to start with.

Another disadvantage of closed claims is the lack of data recency, which refers to the freshness or timeliness of the data in a system. Most commercial closed claims sources have a lag time of 6 months. The latest available Medicaid data from the Centers for Medicare and Medicaid Services has a 3-year lag time, and Medicare data has a 2-year lag. Current patient data are essential for accurate diagnosis, treatment decisions, and health trend monitoring. The US Food and Drug Administration frequently approves medication with postmarketing commitments that show a serious risk related to the use of the drug or that identify unexpected serious risks using real-world data sets. Therefore, the agency and public may have to wait several years for a pharmaceutical company to use commercial closed claims to conduct the research, when the sample size is large enough to analyze its recently approved medication. Most pharmaceutical companies are eager to show the advantage of their product to providers and payers once their medication is on the market, and closed claims may delay these studies.

Closed claims are rarely nationally representative. If a closed-claim feeder was a payer predominant in certain areas of the US, the results will be biased toward the populations represented in those regions. This will be true if the closed-claim feeder was a collection of employers providing employee health insurance data clustered in certain regions of the US. Since demographics, socioeconomic status, and health status change are not randomly distributed across the country, the estimates will not be nationally representative. Among the closed claims, the most nationally representative data sets in terms of geography are the government data sets such as Medicare, Medicaid, and Veterans Affairs (VA) of the US Department of Defense. However, Medicare is mostly representative of ages over 65, Medicaid is representative of the economically disadvantaged population, VA is representative of mostly males.

Open claims, on the other hand, are captured through practice management systems (the information systems that manage medical practices’ scheduling, billing, and other internal functions), “switches” or “clearinghouses” (the companies that route claims from healthcare providers to US insurers), or pharmacy benefit managers (companies that provide the link between pharmacies and insurance companies). Kythera, Komodo, IQVIA’s Longitudinal Prescription Data (LRx), and Medical Claims Data (Dx) are some of the examples of open claims databases. The recency of open claims is a key differentiator. These organizations often receive claims information within days of a patient’s medical or pharmacy encounter; as such, open claims provide a near-real-time view of patient activity. Most open claims data are updated daily or weekly and provide the most up-to-date information about the care.

**Figure 2.**
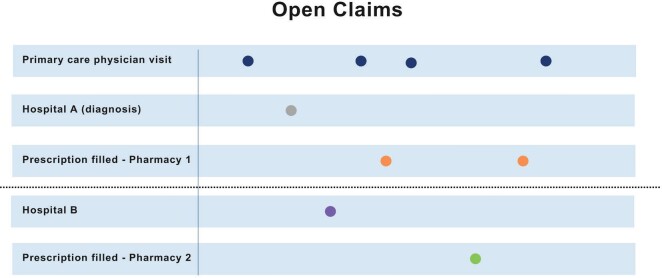
Patient’s Journey in Open Claims

Open claims data provide a glimpse into the patient journey across several data sources over an open-ended period. Since open claims data contain information from a variety of healthcare settings and do not rely on a patient maintaining the same insurance plan or job, the patient can be studied over a longer period. Thus, if Patient A had 2 visits to a primary care physician under Insurance A and 2 others from Insurance B, closed claims that capture the information from Insurance A would not include the last 2 visits since the enrollment with that insurer ended. In open claims, all of these claims would be captured (**Figure 2**).

Open claims include much of the same information content as closed claims—diagnosis codes, procedure codes, and drug identifiers. These data sets can be used for recent launches of medication and market updates, burden-of-disease analysis, comparative effectiveness studies, managed market impact analysis, physician targeting and messaging, source of business, diagnosis allocation, and patient journey. These data sets, relative to closed claims data, are significantly larger (some can cover >300 million patients) and nationally representative, and contain more variables for robust statistical analysis. For example, open claims can capture cash payment information and can be used to analyze such transactions not covered by plans. A recent study showed that more than 20% of the statin claims were missing in a closed claims file because payments were made by an alternate third party program, in cash, or with coupons or discount cards.^7^

Further, closed claims data do not include clinical metrics, which are necessary for robust estimates in outcomes research studies. Disease severities are usually proxied by several comorbidity indices, which cannot capture the true severity of a disease.^8^ In a cloud environment, open claims can be deterministically (not probabilistically) linked with other data sets such as EHR or doctors’ charts and de-identified to enhance the capability of the data set. We can analyze how severity plays a role on disease burden, treatment choices, initiation of treatment, and provider and patient behavior with linked data sets.

However, open claims have several challenges. Therefore, before deciding to use open claims data, researchers should ask several questions:

- Will they have access to raw data, or will they use the data on a platform? Platforms do not give enough flexibility to model typical HEOR. Moreover, assumptions of the query tools can be hidden and not validated in published research. Working on open claims requires significant computing power and knowledge to process because the application of these claims to outcomes research is new.
- What is the average longitudinal patient view? Observing patients over several years is important for HEOR (baseline, identification, follow-up times); therefore, marketed sample sizes can be misleading.
- What is the data lag time? Open-source databases from pharmacies, practice management systems, and clearinghouses usually have a 1- to 3-week lag time. If the vendor’s data are fed from payers as well, that portion would typically have a 5- to 6-month lag time. The payers will need to deliver reconciled data, which can take several months to engineer.
- What are the rates of missing values for the key variables? If there is no mandate on the variables that need to be filled in, and if those variables are crucial for the research, the sample size on the final model will be much smaller than the initial sample size.
- What are the linkage capabilities with the data? If the research requires additional variables, such as lab data, race and socioeconomic status variables, vital statistics, survival information, or cause of death, is it possible to link the open claims file in a cloud space with other data sets, allowing for additional variables in your research?
- Is it possible to replicate the study with closed claims for validation purposes? Open claims will capture patient visits to different doctors, hospitals, and testing centers. All these actions will be captured, regardless of their insurance provider, as long as the providers use the same clearinghouses. Although it is very rare for providers to change their clearinghouses, if a provider changes the clearinghouse that the patient frequently visits or if the patient visits different provider that belongs to a different clearinghouse that does not feed the open claims, those interactions will be missed. Changing a clearinghouse is an infrequent occurrence for providers, as changing clearinghouses can be a time-consuming process that involves updating systems and reestablishing connections with insurance companies. A recent survey showed that 30% of patients, mostly younger ones, have changed providers in 2021. Therefore, it is often difficult to know what percentage of interactions are captured in open claims. Claims-level completeness can also be time dependent or disease dependent. Nearly all open pharmacy claims are observable within several days. Among medical and other claims that accrue, the majority are expected to be available within 21 days. One objective of this article is to compare healthcare utilization of selected diseases between closed claims and open claims to determine if missing interactions affect the estimates.
- Is there any duplication in the data set? Open claims data has the possibility of duplication. Since the claims are captured from the back-and-forth between the provider and the payer from the claim adjudicator as well as from revenue cycle managements and directly from providers, it is possible to see the same person, same procedure, and same dates with multiple records in the source but with different claim IDs on the record. This would create an overestimation of healthcare utilization and costs. In terms of estimating and incidence of prevalence of the disease, the absence an enrollment file in open claims could also be problematic. Although closed claims provide an enrollment file that can be used to establish a denominator, open claims are more like EHR data in that they do not carry enrollment information. As such, the relevant denominator must be estimated through, for example, evidence of activity (ie, any patient on whom activity is observed within a specific time is included in the denominator). To avoid biases due to underinclusion of healthy individuals, definitions of activity that are more sensitive rather than more specific are recommended.^9^ For a particular patient, the eligible person-time can be established similarly, where activity indicates that a patient’s information would be expected to be captured, and lack of activity would exclude that patient’s person-time.

**Figure 3.**
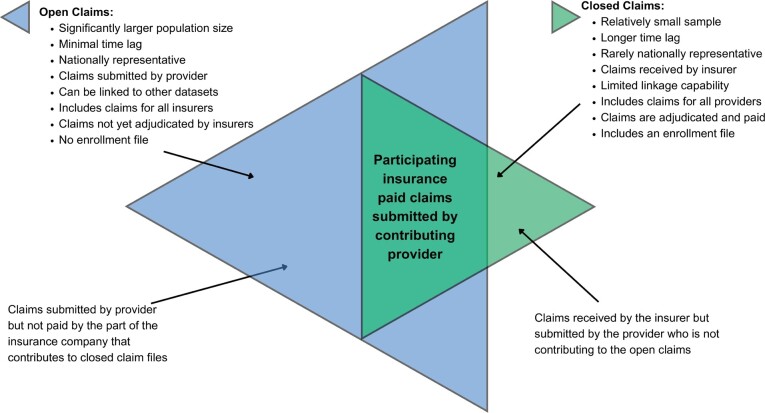
Open vs Closed Claims Adapted from: Franklin JM, Lin KJ, Gatto NM, et al. *Clin Pharmacol Ther.* 2021;109:816-828.

**Figure 3** summarizes the advantages and disadvantages of open and closed claims. If the issues related with open files are resolved, open claims would significantly contribute to health outcomes research as they provide larger, more detailed, and more recent information than closed claims. In this study, we used an open claims file, applied an algorithm to remove possible duplications, and compared healthcare utilization estimates ranging from common to rare for 18 different diseases with those using closed claims data.

## METHODS

### Data Sets

We used 2 closed claims (PharMetrics® Plus, MarketScan®) and 1 open claims file (Kythera). PharMetrics® Plus and MarketScan® data have been used in many outcomes research studies. Kythera is a relatively new open claims data set. It allowed us to work with raw data; had a long patient view, only a 1-week lag time, and no missing values on our variables; and could be linked with other EHR data sets in a Datavant environment.^10^

### MarketScan® Data Set

MarketScan® databases are constructed by collecting data from employers, health plans, and state Medicaid agencies who are our customers and have agreed to be data contributors. It is a closed claims data set. Data comprise service-level claims for inpatient and outpatient services and outpatient prescription drugs. All claims have been fully paid and adjudicated, and financial, clinical, and demographic fields are all standardized. Drug detail (eg, therapeutic class, therapeutic group, manufacturer’s average wholesale price, a generic product identifier) and clinical detail (eg, disease episode grouper) are available. A unique enrollee identifier is assigned to each individual in a MarketScan® database. Data are collected for the MarketScan® annual database releases when nearly 100% of claims have been paid; this removes the need for completion factors and helps improve the reliability and accuracy of the data. In the most recent full data year, MarketScan® data sets contain healthcare data for more than 39.7 million covered individuals.

### PharMetrics® Plus Data Set

PharMetrics® Plus contains fully adjudicated medical and pharmacy claims data for approximately 40 million patients in any given recent year across all 50 US states, with an average length of health plan enrollment of 36 months. Commercial insurance is the most frequent plan type captured (the database is generally representative of the under-65, commercially insured population in the US), but other types can also be found, including Medicare, and self-insured employer groups (as managed by a health plan). The database contains information on patient demographics, plan enrollment, inpatient and outpatient medical claims, and outpatient pharmacy claims.

### Kythera Data Set

Kythera is an open claims database, updated daily, containing over 310 million patients and 9.7 billion healthcare claims, with 79% coverage of all US patients. The data cover 3 million practitioners, 400 000 organizations, and 1.2 million facilities. Of 310 million patients, 140 million have closed claims. The closed claims portion of Kythera has a lag time of 2 to 3 months. When a claim is transacted with a payer, Kythera captures the back-and-forth between the provider and the payer from the claim adjudicator—the technology vendor during the claim communication process. A small portion of transactions are also captured from revenue cycle management (point of sale) systems and directly from providers. Kythera’s transactional data covers 70% to 95% of all medical events and 50% to 65% of all prescription events in the US since 2016. Reference data (eg, diagnosis, procedure, drug) is acquired from industry standard sources such as the Food and Drug Administration and American Medical Association. Directories (eg, provider, payer) are built in-house from a combination of public and private sources and by integrating metadata from our transactional data. This approach ensures the most accurate data and a tight fit between the transactional and reference/directory information.

Understanding how the procedure and episodes get billed is key to mapping the patient journey because data scientists know what to look for in the claims, such as inpatient, outpatient, pharmacy, anesthesia, and physician information. Using every claim possible enriches a data set with additional variables and ensures to build events for multiclaim healthcare encounters. Open claims data sets use several sources to make sure that they have all the locations for health care.

For example, Dr. X utilizes a primary facility location (123 Main Street) for specific claims, while for other claims, the address may be a post office box or an alternative address, depending on the payer. Given that the facility address is known, it may be inferred that the remaining addresses pertain to the billing office. In the scenario where a female patient, born in 1964, was attended to by Dr. X and the billing information provided included a post office box, it is possible to establish a connection between this patient and the facility address. Consequently, the address where the medical care was administered can be accurately entered.

**Figure 4.**
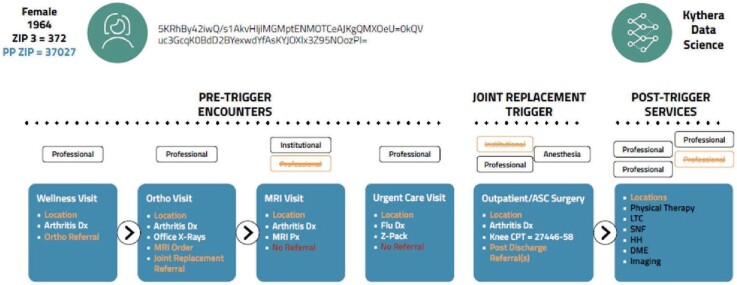
Typical Claims in Kythera Data

**Figure 4** illustrates the process through which the patient journey is mapped from a combination of multiple claims. The initial encounter consisted of a primary care wellness visit, during which a diagnosis of arthritis was established. Subsequently, the primary care provider initiated a referral for an orthopedic consultation (in orange). The orthopedic doctor confirmed the diagnosis and proceeded to conduct x-rays in their office. Additionally, they ordered magnetic resonance imaging (MRI) and afterward made a referral for joint replacement. The initial two bills are for professional expenses related to data processing. There is no institutional claim for the visits. Following MRI, there are two claim types: one institutional and the other professional. Therefore, there are two chances to identify MRI. This patient, then, had an urgent care visit unrelated with joint replacement. For the episode analysis, that claim would be dropped. A patient receiving a joint replacement may trigger 4 to 7 claims. However, it is sufficient to have just one claim to confirm that the patient underwent the procedure. If all the claims pertaining to institutions, professionals, and anesthesia are included in the data set, the data will comprehensively encompass all relevant information on the process, ranging from referrals to the actual execution of the procedure. Nevertheless, even in the scenario where there is only a single claim for anesthesia, it is still possible to extract data regarding the type of operation, the location of the procedure, the entity responsible for payment, and the recipient of the service.

The construction of the patient journey in Kythera effectively detects appropriate referrals. The other data sets, following common patient logic, list last the specialist doctor seen as a referring provider. In the context of joint replacement, it is possible to consult either the radiologist who conducted the MRI or the physicians in an urgent care facility as referring practitioners, provided that they were the most recent specialists visited. The Kythera data ensures that physicians who visit MRI facilities or urgent care centers are not erroneously classified as referral doctors, as shown in red in **Figure 4**. The claim submitted to urgent care was for influenza, and it is important to note that the MRI radiologist cannot be categorized as a referral. Thus, it applies the business rule to correctly identify referrals.

The data do not rely on a single claim to understand a healthcare encounter, and since the entire data set contains the patient journey by stage and age/gender bands for half of the population seeking care every year, the likelihood of accuracy to identify the patients that have full records from the open claims is significantly high.

All of these data sets are de-identified and compliant with the Health Insurance Portability and Accountability Act (HIPAA).

We used MarketScan® and PharMetrics® Plus data as closed claims for 18 diseases using 2022 data as an identification period. After identifying each disease, we calculated the percentage of patients with hospitalization, ED, and outpatient visits. Patients were continuously enrolled from January 2021 to December 2022.

Since enrollment data available from these 2 different closed claims capture more than 80 million of the US population (dropping <5% possible overlap between those 2 claims), for each disease by age, gender and stages (proxied by comorbidity scores, prediagnosis healthcare costs and utilization), one can match these patient journeys to open Kythera claims to identify the patients with closed claims. This would minimize the bias due to possibility of missing claims resulting from the open structure of the data set.

In the Kythera data, to identify possible duplications within the data set we matched patients with different IDs on gender, state, birth year, Charlson comorbidity score, comorbid disease score, total inpatient, ED, and physician visits 6 months prior to the index date. Among the patients that matched on these variables, one was considered for final analysis and the rest would be dropped. The same outcome variables were created with the data.

We used standardized differences to compare the estimates from the closed claims data set to the open claims data set. Standardized differences are important to distinguish practical (ie., clinical) from statistical significance. For example, some variables may be statistically significant (as indicated by *p* values) due to a large sample size, although the practical significance is small (as indicated by standardized differences).

The standardized difference was calculated as:

$$d=\frac{\left({\bar{X}}_{a}-{\bar{X}}_{b}\right)}{\sqrt{S_{a}^{2}+S_{b}^{2}/2}}$$

for continuous variables; and

$$d=\frac{\left(p_{a}-p_{b}\right)}{\sqrt{\left[p_{a}\left(1-p_{a}\right)+p_{b}\left(1-p_{b}\right)\right]/2}}$$

for dichotomous variables, where ${\overset{―}{X}}_{i}$ is the average value for patients in group *i*, *S_i_* is the corresponding standard error, and *p_i_* is the percentage of patients in group *i*.^11^ Outcomes were compared using standardized differences.

## RESULTS

The results are shown in **Table 1**. The sample size of open claims data was 10 to 65 times larger than closed claims data, with 613 823 patients were identified with alcohol dependence in open claims data compared with only 36 287 patients in the closed claims data in MarketScan® and 14 174 patients in PharMetrics® Plus. Comparing healthcare utilization, we found comparable results: 19.28% of the patients in Kythera were hospitalized with a diagnosis of alcohol abuse, which was between the percentages from the closed claims databases (16.76% and 21.69%).

**Table 1.** Sample Sizes and Overall Ratio from Open vs Closed Claims

	**Kythera**					**MarketScan®**					**PharMetrics® Plus**				
**Diagnosis**	**n**	**Inpatient (%)**	**ED (%)**	**Outpatient (%)**	**LOS (days)**	**n**	**Inpatient (%)**	**ED (%)**	**Outpatient (%)**	**LOS (days)**	**n**	**Inpatient (%)**	**ED (%)**	**Outpatient (%)**	**LOS (days)**
Alcohol dependence	613 823	19.28	37.16	98.04	3.01	36 287	21.69	35.71	96.45	3.87	14 174	16.76	30.10	95.58	2.29
Ankylosing spondylitis	69 811	6.29	16.50	98.36	0.81	7875	6.03	24.86	99.06	0.45	2495	5.13	16.95	98.88	0.40
Bladder cancer	142 627	12.59	20.06	98.98	1.77	3883	11.82	25.83	99.38	0.97	2201	13.86	22.49	99.05	1.43
Cirrhosis	24 481	8.50	16.68	98.46	1.17	1509	9.34	25.51	99.54	0.88	582	7.39	19.07	99.48	1.37
Chronic kidney disease	2 447 280	19.16	27.54	99.21	3.85	82 017	15.64	27.43	99.02	2.03	35 926	14.45	22.70	98.72	1.97
Crohn’s disease	254 126	9.01	19.39	98.09	1.22	26 586	10.28	26.19	98.65	0.96	9075	9.31	19.79	98.28	0.88
Cystic fibrosis	22 704	9.30	14.24	96.47	1.59	1649	13.46	24.01	99.03	2.08	525	11.05	17.33	98.29	1.14
Endometriosis	187 539	5.32	22.95	98.56	0.40	31 405	6.17	30.43	99.18	0.36	8461	4.72	21.34	98.83	0.30
Hepatitis B	98 414	5.88	12.53	97.95	0.94	8175	4.24	14.45	95.11	0.43	2411	3.94	9.00	94.53	0.52
Leiomyoma of uterus	460 347	5.55	20.22	98.75	0.57	92 494	4.37	24.58	98.74	0.30	21 672	3.74	15.85	98.33	0.28
Lupus	51 794	8.46	20.60	98.75	1.26	5333	7.43	27.66	98.93	0.72	1500	7.47	20.00	98.87	0.85
Multiple sclerosis	231 609	8.75	17.16	97.93	2.63	19 881	7.08	23.99	98.80	0.62	6182	5.95	17.26	98.56	0.58
Myositis	94 037	9.99	21.12	98.67	1.66	16 338	6.33	24.95	98.07	0.64	5750	5.17	16.28	97.74	0.60
Opioid dependence	785 702	13.59	30.77	98.17	2.06	24 773	16.77	34.63	98.11	3.14	9190	13.88	28.05	97.64	1.94
Rheumatoid arthritis	267 057	7.73	17.34	98.94	1.02	25 622	6.05	23.17	99.55	0.48	8994	5.85	16.47	99.29	0.45
Schizophrenia	334 399	25.48	36.64	97.90	9.38	4357	29.97	39.84	97.13	6.93	1246	26.48	36.12	96.31	5.24
Type 1 diabetes mellitus	611 653	12.06	20.88	98.08	2.39	53 499	10.42	23.87	98.48	0.97	17 725	8.72	17.77	98.38	0.83
Ulcerative colitis	274 323	9.55	19.14	97.88	1.52	31 775	8.59	23.93	98.17	0.83	10 504	7.60	18.30	97.83	0.78

Abbreviations: ED, emergency department; LOS, length of stay.

When we compared healthcare utilization before diagnosis, we found comparable results. The percentage of hospitalized alcohol abuse patients identified in open claims data (19.28%) was between the percentages found between the closed claims PharMetrics® Plus and MarketScan® data (16.76% and 21.69%, respectively). Of the alcohol abuse patients in the ED, 37.16% were identified in open claims, which was between the percentages found from PharMetrics® Plus and MarketScan® (30.10% and 35.71%, respectively), and 98.04% of the alcohol abuse patients identified were outpatients in open claims, which was between the percentages found in the closed claims PharMetrics® Plus and MarketScan® data (95.58% and 96.45%, respectively).

Similar results were found in other dependence disorders, such as opioid dependence (785 702 patients were identified with opioid dependence in open claims data vs 24 773 patients in MarketScan® and 9190 in PharMetrics® Plus).

There were similar results in the comparison between the claims regarding healthcare utilization. We found that for outpatient data only, the difference was remarkably close (98.17%, 98.11%, and 97.64%, for Kythera, MarketScan®, and PharMetrics® Plus, respectively).

Similar trends were found in other chronic diseases such as chronic kidney disease, cirrhosis, Crohn’s disease, ulcerative colitis, lupus, multiple sclerosis, myositis, and rheumatoid arthritis. For outpatient data, the difference between them was remarkably close. Furthermore, in the other aspects of healthcare utilization data (such as inpatient and ED visits), the open-claim percentage will always be between the closed claims percentages.

In childhood diseases such as type 1 diabetes mellitus, we found a similar trend: 611 653 patients were identified with Kythera, whereas 53 499 patients were identified with MarketScan®, and 17 725 were identified with PharMetrics® Plus, and 20.88% of identified patients had ED visits, which was between the percentages found in the 2 closed claims data sets (17.77% and 23.87 % for PharMetrics® Plus and MarketScan®, respectively).

We compared our estimates from closed claims with open claims using standardized differences. Particular estimates from MarketScan® and PharMetrics® Plus data were compared with open claims Kythera data separately. In statistics, standardized differences of 0.2 to 0.5 are considered small, 0.5 to 0.8 are considered medium, and values greater than 0.8 are considered large.^12^ All of the differences in the healthcare utilization estimates were negligible between the closed claims and Kythera open claims file (**Table 2**).

**Table 2.** Standardized Differences between Open Claims (Kythera) and Closed Claims Data Sets

	**Kythera vs MarketScan® (Std. Diff.)**				**Kythera vs PharMetrics® Plus (Std. Diff.)**			
Diagnosis	Inpatient	Emergency	Outpatient	LOS	Inpatient	Emergency	Outpatient	LOS
Ankylosing spondylitis	0.0250	0.2841	0.3120	0.1605	0.1195	0.0178	0.2131	0.1769
Bladder cancer	0.0395	0.1806	0.2777	0.1572	0.0610	0.0799	0.0365	0.0652
Cirrhosis	0.0570	0.2962	0.4690	0.0874	0.0843	0.0900	0.4106	0.0576
Chronic kidney disease	0.1353	0.0031	0.1171	0.1648	0.1866	0.1422	0.2700	0.1694
Crohn’s disease	0.0803	0.2145	0.1939	0.0769	0.0196	0.0141	0.0587	0.0972
Cystic fibrosis	0.2297	0.3549	0.7261	0.1053	0.1055	0.1286	0.4082	0.0968
Endometriosis	0.0864	0.2119	0.3094	0.0296	0.0704	0.0514	0.1146	0.0877
Hepatitis B	0.1893	0.0907	0.4946	0.1911	0.2321	0.2042	0.4600	0.1534
Leiomyoma of uterus	0.1388	0.1387	0.0041	0.1771	0.2278	0.1633	0.1619	0.1799
Lupus	0.0779	0.2136	0.0861	0.1517	0.0746	0.0206	0.0534	0.1125
Multiple sclerosis	0.1265	0.2321	0.3035	0.2732	0.2290	0.0037	0.2028	0.2720
Myositis	0.2736	0.1192	0.2061	0.2261	0.3924	0.1764	0.2958	0.2257
Opioid dependence	0.1366	0.0969	0.0164	0.1782	0.0138	0.0722	0.1433	0.0195
Rheumatoid arthritis	0.1449	0.2002	0.4713	0.1889	0.1651	0.0343	0.2205	0.1945
Schizophrenia	0.1239	0.0749	0.1776	0.0901	0.0288	0.0124	0.3213	0.1524
Type 1 diabetes mellitus	0.0906	0.0950	0.1311	0.2115	0.1990	0.1102	0.0923	0.2279
Ulcerative colitis	0.0645	0.1568	0.0844	0.1628	0.1378	0.0305	0.0131	0.1714

Abbreviations: ED, emergency department; LOS, length of stay; Std. Diff., standardized difference.

## DISCUSSION

RCTs are deemed the ideal evaluation technique for health care and treatments. However, randomization is often not feasible or permissible, such as in the following circumstances:

- Treatment is in its early stages and may need frequent adjustments to perfect operation and delivery.
- Enrollment demand is minimal, which may occur when few patients express consent to potential treatment or when diversion of a subset of potential patients to control status may be unacceptable.
- Physicians have ethical qualms about denying treatment to those perceived to be in need.
- Schedules and budgets are limited, as RCTs often require an extensive management process that require a large amount of time and money.
- RCT may be less generalizable to the population of interest.
- The integrity of the evaluation may be easily threatened, especially through failure of treatment or control group members to follow protocol, morbidity or mortality, or other reasons for dropping out of the evaluation.^13^

Under these circumstances, or to see if the results from RCTs are similar in a real-world setting, observational studies would be the design of choice. Some statistical techniques are available to adjust for the heterogeneity of real-life populations in real-world studies.^14^ If the adjustment is done correctly, results from observational studies are shown to be statistically similar to the results from RCTs.^1,15^

Due to the large sample size, the availability of more variables, the possibility of linkage to other claims that capture clinical and quality measures, and the recency of the data, the merits of using open claims have become increasingly recognized over the years as their application grows. Nevertheless, the possibility of missing claims and duplication may make researchers suspicious of the results of open claims studies. However, as shown in **Table 1**, with appropriate data cleaning and engineering, open claims data sets can provide statistically similar results to closed claims files. Until the body of literature builds as in closed claims data, validation of the results from open claims with the results from closed claims would be the sensitive approach.

## CONCLUSION

Open claims data, with a bigger sample size and their recency, provide essential advantages for health outcomes research studies. With open claims, there is a chance of missing claims due to data structure, but once cleaned, they can provide estimates close to closed claims. Compared with closed claims data regarding healthcare utilization, open claims data were found to be close to the range, regardless of the etiology of the disease. Therefore, especially for new medications and rare diseases, open claims data can provide information much earlier than closed claims, which usually have a small sample size and have a time lag of 6 to 8 months.
